# Stationarity and cycles in the energy consumption in the United States

**DOI:** 10.1007/s11356-024-32248-7

**Published:** 2024-02-15

**Authors:** María José Presno, Manuel Landajo

**Affiliations:** Department of Applied Economics, University of Oviedo, Avda del Cristo s/n, Oviedo, Spain

**Keywords:** Energy consumption, Stationarity testing, Nonparametric, Panel, Cycles, Concordance

## Abstract

The purpose of this paper is twofold: analyzing stationarity of energy consumption by source in the United States and studying their cycles and pairwise synchronization. We study a panel of nine time series of monthly energy consumption for the period 1973–2022. Four of the series (namely coal, natural gas, petroleum, and nuclear electric power consumption) are non-renewables, whereas the remaining ones (hydroelectric power, geothermal, biomass, solar, and wind energy consumption) are renewable energy sources. We employ a nonparametric, panel stationarity testing approach. The results indicate that most of the series may be trend-stationarity, with nuclear and geothermal energy consumption being the only exceptions. Additionally, a study on potential cycles in the series of energy consumption by source is carried out, and subsequently we analyze pairwise concordance between states of different energy sources and between states of energy sources and the business cycle. Significant correlations are detected in the latter analysis, which are positive in the case of fossil fuel sources and negative for two renewable sources, namely geothermal and biomass energy consumption.

## Introduction

The relationship between economic activity and energy consumption has been widely studied. Shocks in energy consumption, such as those induced by an increase in energy prices, tend to spill over to the economy and to variables like real interest rates, inflation, and real exchange rates.

This paper aims primarily at analyzing stationarity of energy consumption by source in the United States (US). This topic is important in several fields, including model estimation, forecasting, and policy design. This way, energy promoting policies could be effectively implemented in those cases where the effects of shocks are permanent. On the contrary, when the shocks on a specific energy source are transitory in nature, conducting interventions in that sector would be unnecessary. Also, since energy is correlated with both environmental quality and economic activity, the stochastic characteristics of energy consumption will tend to spill over to other macroeconomics variables.^1^

In this paper we address energy consumption from the standpoint of energy sources. The analysis is carried out at three different levels. First, we consider a panel of 7 energy consumption sources (namely coal, natural gas, petroleum, nuclear electric power, hydroelectric, geothermal, and biomass). Secondly, we shall study two subpanels separately, including, respectively, energy consumption coming from non-renewable and renewable sources. A differentiated analysis of non-renewable and renewable sources is relevant since the renewable energy segment has become increasingly important because of the growing focus on reducing greenhouse gas emissions that strongly contribute to climate change, as well as other factors like petroleum shortages, restrictions caused by the war in Ukraine, volatile energy prices, and other energy security concerns. Finally, we conduct a disaggregated analysis of energy consumption, individually for each energy source (including wind and solar energy consumption). This separate analysis will allow us to determine more specific incentive and energy conservation policies for each source of energy consumption.

From a methodological standpoint, one problem that plagues both stationarity and unit root testing is their sensitivity to incorrect specification of the deterministic components of the process. The consequences of this sensitivity to misspecification are potentially serious. For instance, in stationarity testing, it causes spurious unit roots to be detected in the series. A solution to that problem comes from nonparametric methods, that provide asymptotically correct tests without requiring previous specification of a parametric model. In this paper we shall rely on the nonparametric stationarity testing framework originally proposed by Landajo and Presno (2013) and extended to panel analysis by Presno et al. (2022).

Additionally, in the second part of the paper, we carry out an analysis to assess the potential existence of cycles in the time series analyzed. We begin by examining the degree of synchronization among the states identified for the various sources of energy consumption. The analysis will be extended to study synchronization between each source and the business cycle as defined by the National Bureau of Economic Research (NBER). To our knowledge, this is the first analysis to include both the cycles in the various sources of energy consumption and the synchronization among them and with the business cycle.

In summary, the contribution of the paper is twofold: first, we analyze stationarity of energy consumption by source in the United States, employing a nonparametric stationarity testing framework. The study is carried out at three different levels (respectively, panel of all the sources, panels of renewable and non-renewable sources, and individual series). This analysis makes it possible to devise more effective policies for each specific source. Secondly, we study the potential presence of both cycles in the series of energy consumption by source and pairwise synchronization between pairs of sources and (because of the correlation between energy consumption and economic activity) with the business cycle.

The rest of the paper is structured as follows: Section "Literature review" includes a literature review and Section "Methodology" outlines the methodology we rely on. The main results appear in Section "Empirical analysis". A summary of conclusions, some final remarks and several potential research avenues is included in Section "The dataset".

## Literature review

A growing literature studies the stochastic properties of energy consumption time series. Hsu et al. (2008) include a compilation^2^ of earlier results from univariate unit root tests in this field. Altinay and Karagol (2004), Lee and Chang (2005), and Lee (2005) are among the pioneering papers in the field of energy consumption that employed univariate unit root tests including structural breaks. More recent advances, aiming at overcoming the problems of low power that plague univariate unit root testing in short time series, have led to extending classical univariate unit root testing to the analysis of panel data. Using panel unit root testing, Narayan and Smyth (2007) and Chen and Lee (2007) find strongly significant evidence of stationarity in per capita energy consumption. On the contrary, Hsu et al. (2008) explore energy consumption stationarity in several geographic areas, finding evidence of integratedness. Thereafter, an abundant stream of papers has researched stationarity in consumption from various perspectives (including source, sector, and country), employing a wide range of techniques (e.g., unit root, stationarity, and panel tests), and relying on various model specifications (linear, nonlinear, and flexible Fourier, among others). Along this line, Aslan and Kum (2011) analyze the case of Turkey by using both linear and non-linear unit root tests to investigate stationarity of energy consumption, disaggregated into seven sectors (namely residential, industrial, transportation, agricultural, non-energy uses & other, final energy consumption, and cycle & energy). Also for disaggregated energy consumption in Turkey, Erdogan et al. (2020) employ conventional unit root and Fourier panel stationarity tests, concluding that disaggregated energy use in the country is nonstationary. Yilanci and Tunali (2014), using a flexible Fourier Lagrange Multiplier unit root test, examine the properties of per capita energy consumption for more than a hundred countries, finding stationarity in about 25% of them. Similarly, Hasanov and Telatar (2011) study the stochastic behavior of per capita total primary energy consumption for a large dataset of 178 countries by applying tests that allow for structural breaks and nonlinear adjustments in the series. Their results indicate that, when all those features of the data are properly modeled, most of the series analyzed are classified as stationary processes. Kula et al. (2012) addressed the case of per capita electricity consumption in OECD countries by using a Lagrange Multiplier unit root test that endogenously determines structural breaks, obtaining evidence of stationarity in almost all the nations considered in their study.

In the field of non-renewable sources, Solarin and Lean (2016) applied both linear and nonlinear stationarity tests to examine oil consumption in 57 countries, finding nonlinear patterns in nearly 50% of the countries. Shahbaz et al. (2014) (for a 48-country panel) and Cai and Magazzino (2019) (for the G7 states) analyzed natural gas consumption, reporting stationarity in most of these time series. Zhu and Guo (2016) also conclude that per capita nuclear energy consumption, in a panel of 27 countries over the period 1993–2013, is stationary.

Wang et al. (2016) address Japan´s consumption of non-fossil energy from various sources. Their conclusions emphasize the different patterns of nuclear energy (that, according to their results, presents a unit root) and renewable energy consumption (which they classify as stationary). Other papers have focused exclusively on renewable sources. Among them, Lean and Smyth (2014) apply both univariate and panel Lagrange Multiplier unit root testing to hydroelectricity consumption in 55 nations, finding evidence of stationarity in most of them. Gozgor (2016) and Demir and Gozgor (2018) analyze renewable energy consumption in both developing and developed countries; their results also show stationarity in most of them. Evidence of stationarity in the renewable-to-total ratio in electricity consumption, for a panel of 90 countries, is also found by Tiwari and Albulescu (2016) by employing a flexible Fourier stationarity test. Cai and Menegaki (2019), for their part, study convergence of clean energy consumption for 35 emerging and OECD countries. Their analysis, which relies on panel unit root testing with both instant and gradual changes allowed for in their models, stresses the importance of taking structural changes into account.

Focusing specifically on the case of the US, several papers have analyzed data from various states and/or sources of energy by employing a large variety of techniques. Analyzing data from the 50 US states, Apergis and Payne (2010) apply two-break unit root tests to the petroleum consumption series and conclude stationarity in most states. Apergis et al. (2010a) use panel stationarity and unit root tests with endogenous structural breaks to study coal consumption in a panel of US states^3^, finding that this variable is integrated of order zero. The same techniques, in addition to a battery of panel tests that do not incorporate structural breaks, were employed by Apergis et al. (2010b) to investigate natural gas consumption. This variable is also studied by Aslan (2011), who (by employing a nonlinear unit root test) finds nonlinearity in most of the states and similar percentages of stationary and nonstationary series.

For their part, Lean and Smyth (2009) research the long memory properties of disaggregated petroleum consumption (covering transportation, residential, industrial, electric power, and commercial sectors). They report that conclusions generally depend on the specific sector under study, emphasizing the need to distinguish among the various forms of consumption. Along this line, Apergis and Tsoumas (2012) extend the analysis to disaggregated coal, fossils, and electricity retail consumption. Gil-Alana et al. (2010) focus on the analysis of long memory in energy consumption by the US electric power sector. Golpe et al. (2012) study persistence in quarterly observations of natural gas consumption in the US. More recently, Fallahi (2019) analyses stationarity and persistence in sectoral energy consumption in the US by using bootstrap and subsampling confidence intervals. Adekoya (2020) has studied the long memory behavior of energy consumption by source; his analysis considers the effects of seasonality and structural breaks, finding that both are strongly significant and concluding that in most cases their inclusion in the models alters the degree of persistence. Mishra and Smyth (2014) analyze monthly US natural gas consumption by employing a GARCH unit root test with structural breaks, showing the importance of accounting for conditional heteroscedasticity when testing for unit roots in energy consumption series with high-frequency data.

In the field of renewable energies, Apergis and Tsoumas (2011) research the stochastic characteristics of disaggregated geothermal, biomass, and solar energy consumption in the US. Aydin and Pata (2020) also study disaggregated renewable energy consumption data, using a wavelet-based unit root test that takes both smooth structural changes and frequency components into account. Employing long memory techniques, Barros et al. (2012; 2013) research US renewable energy consumption, both total and disaggregated by source. Lee et al. (2021) test for the persistence of shocks on renewable energy consumption, both at the federal level and for the 50 US states plus the District of Columbia. They use a quantile unit root test that leads them to conclude that the unit root hypothesis is rejected in more than half of the US states.

Table 1 below shows a summary of papers that have investigated energy consumption in the U.S., classified in three categories according to the variable under study (respectively, overall consumption, non-renewable, and renewable sources).

**Table 1:** Summary of literature

Author	Period. Variable	Technique	Conclusion
Overall consumption			
Narayan and Smyth (2007)	Annual data. 1979–2000 Per capita energy consumption	Univariate unit root test. Panel unit root testing for groups of countries	Non-stationarity
Chen and Lee (2007)	Annual data. 1971–2002 Per capita energy consumption	Univariate and panel stationarity tests with structural breaks	Non-stationarity
Hsu et al. (2008)	Annual data. 1971–2003 Per capita energy consumption	Univariate unit root and stationarity tests. A panel SURADF test	Non-stationarity
Gil-Alana et al. (2010)	Monthly data. 1973:01–2009:05. Electric power sector	Fractional integration with structural break	Fractional integration
Hasanov and Telatar (2011)	Annual data. 1980–2006 Per capita energy consumption	Univariate unit root tests (ADF, a test allowing for non-linear adjustment, and a test combining smooth transition and threshold autoregressive methodologies)	Stationarity under any of the three tests
Kula et al. (2012)	Annual data. 1960–2005. Electricity consumption per capita	Univariate unit root tests with up to two endogenous structural breaks	Stationarity
Yilanci and Tunali (2014)	Annual data. 1960–2011 Per capita energy consumption	Fourier Lagrange Multiplier unit root test	Stationarity
Fallahi (2019)	Annual data. 1952–2016 Sectoral energy consumption	Analysis of persistence and stationarity using bootstrap and subsampling confidence intervals	Mixed evidence depending on the sector
Adekoya (2020)	Monthly data. 1973:01–2018:08. Energy consumption by source	Fractional integration. Inclusion of structural breaks and seasonality	Long memory, strong seasonal patterns, and significant structural breaks
Non-renewable sources			
Lean and Smyth (2009)	Monthly data. 1973:01–2008:07. Disaggregated petroleum consumption	Fractional integration	Mixed evidence depending on the sector
Apergis and Payne (2010)	Annual data. 1960–2007 States of the U.S. Petroleum consumption	Univariate two-break unit root tests	Stationarity in most states
Apergis et al. (2010a)	Annual data. 1982–2007 States of the U.S. Coal consumption	Panel stationarity and unit root tests with endogenous structural breaks	Stationarity
Apergis et al. (2010b)	Annual data. 1980–2007 States of the U.S. Natural gas consumption	Panel stationarity and unit root tests with/without endogenous structural breaks	Stationarity (tests with breaks) Non-stationarity (tests without breaks)
Aslan (2011)	Annual data. 1960–2008 States of the U.S. Natural gas consumption	Univariate unit root tests with up to two breaks and nonlinear unit root test	Nonlinearity. Similar percentages of stationary and nonstationary series
Congregado et al. (2012)	Quarterly data. 1973:01–2010:03. Coal consumption	Non-linear specification of an unobserved components model	Persistence
Apergis and Tsoumas (2012)	Monthly data. 1989:01–2009:12. Disaggregated coal, fossils, and electricity retail consumption	Fractional integration including structural breaks	Heterogeneity in results depending on energy type and sector
Golpe et al. (2012)	Quarterly data. 1973:01–2010:03. Natural gas consumption	Non-linear specification of an unobserved components model	Persistence
Shahbaz et al. (2014)	Annual data. 1971–2010 Per capita natural gas consumption	Univariate nonlinear unit root test and panel unit root tests incorporating structural breaks	Nonlinearity and stationarity
Mishra and Smyth (2014)	Monthly data. 1974:04–2013:09. Natural gas consumption	GARCH unit root test with structural breaks	Stationarity
Solarin and Lean (2016)	Annual data. 1965–2012 Total oil consumption	Linear and nonlinear stationarity tests	Stationarity
Cai and Magazino (2019)	Annual data. 1965–2016 Natural gas consumption	Panel stationarity test allowing for both sharp and smooth shifts	Non-stationarity
Renewable sources			
Apergis and Tsoumas (2011)	Monthly data. 1989:01–2009:12. Consumption of disaggregated geothermal, biomass, and solar energy by sector	Fractional integration with and without structural breaks	Mixed conclusions depending on source and sector
Barros et al. (2012)	Monthly data. 1981:01–2010:10. U.S. total renewable energy consumption	Fractional integration	Long memory behavior, with persistence and seasonality
Barros et al. (2013)	Monthly data. 1994:02–2011:10. Hydropower, geothermal, solar, wind, wood, waste, and biofuels energy consumption	Fractional integration incorporating the presence of breaks in the data	Long memory model, with persistence and seasonality
Lean and Smyth (2014)	Annual data. 1965–2011. Hydroelectricity consumption	Lagrange Multiplier family of univariate and panel unit root tests with up to two structural breaks	Mixed conclusions depending on the number of breaks
Tiwari and Albulescu (2016)	Annual data. 1980–2011. Renewable-to-total electricity consumption	Flexible Fourier stationarity test and Fourier ADF unit root test	Non-stationarity (stationarity test) Stationarity (ADF test)
Demir and Gozgor (2018)	Annual data. 1971–2016. Renewable energy consumption	Unit root test with two endogenous breaks	Stationarity
Cai and Menegaki (2019)	Annual data. 1965–2016 Clean energy consumption	Unit root test with both sharp and smooth breaks	Non-stationarity
Aydin and Pata (2020)	Monthly data. 1973:01–2019:09. Renewable energy consumption by source	Wavelet-based unit root test with both smooth structural changes and frequency components	Conclusions depend on source
Lee et al. (2021)	Annual data. 1960–2017 U.S., and 50 States plus Columbia renewable energy consumption	Quantile unit root test with smooth shifts	Stationarity in U.S. consumption and in more than half of the States

## Methodology

The observed data is a panel of *N* time series ${{\varvec{y}}}_{t}=\left({y}_{1,t},..., {y}_{N,t}\right)$ generated by the following process:

1 $${y}_{i,t}={\mu }_{i,t}+ {\theta }_{i}^{*}\left({~}^{t}\!\left/ \!{~}_{T}\right.\right)+{ \varepsilon }_{i,t},$$

$$\mu_{i,t}=\mu_{i,t-1}+u_{i,t};\;t=1,\dots,T;\;i=1,2,\dots,N$$

where ${\theta }_{i}^{*}\left({~}^{t}\!\left/ \!{~}_{T}\right.\right)$ represents the deterministic trend function of the *i*-th component of the panel, and ${{\varvec{\varepsilon}}}_{t} = ({\varepsilon }_{1,t}, ..., {\varepsilon }_{N,t})$ is a zero-mean random vector process which allows both serial dependence and cross-correlation.

The panel testing problem is expressed as:

2 $$H_0:q_i\equiv\frac{\sigma_{i,u}^2}{\sigma_{i,\varepsilon}^2}=0\;\mathrm{for}\;i=1,...,N,\;\mathrm{versus}\;H_1:\sum\nolimits_{i=1}^Nq_i>0$$

Under the null hypothesis (*H*_0_), all the *N* series are trend-stationary, whereas under the alternative (*H*_1_) at least one of the components of the panel has a unit root. The same framework applies to testing for stationarity separately for any individual series in the panel.

Landajo and Presno (2013) developed a univariate nonparametric extension of classical KPSS (Kwiatkowski et al. 1992) stationarity testing. In their proposal, the trend of series ${y}_{i,t}$ is estimated nonparametrically, through an OLS regression of ${y}_{i,t}$ on the elements of a cosine basis. The following estimator is obtained:

3 $${\widehat{\theta }}_{i}\left({~}^{t}\!\left/ \!{~}_{T}\right.\right)={\widehat{\beta }}_{i,o}+\sum\nolimits_{j=1}^{{m}_{T}}{\widehat{\beta }}_{i,j}{\text{cos}}(j\pi t/T)$$

${m}_{T}$ in (3) denotes model complexity. Following Landajo and Presno (2013), we shall use the deterministic rule ${m}_{T}=\left[4{T}^{1/5}\right]$, with $\left[\cdot \right]$ being the integer part function.^4^

From the residuals of the above cosine regression, the raw stationarity test statistic (${\widehat{S}}_{i,T}$) for ${y}_{i,t}$ is computed as follows:

4 $${{\widehat{S}}_{i,T}=\frac{\sum_{t=1}^{T}\left(\sum_{k=1}^{t}{\widehat{\varepsilon }}_{i,k}\right)}{{\widehat{\sigma }}_{i}^{2}{T}^{2}}}^{2}$$

where ${\widehat{\varepsilon }}_{i,k}={y}_{i,k}-{\widehat{\theta }}_{i}\left({~}^{k}\!\left/ \!{~}_{T}\right.\right)$, $k=1,\dots ,T$, and ${\widehat{\sigma }}_{i}^{2}$ is a suitable estimator for the long run variance of ${y}_{i,t}$.

The test statistic for individual series ${y}_{i,t}$ is

5 $${\widehat{Z}}_{i,T}=\frac{{\widehat{S}}_{i,T}-{\mu }_{{m}_{T}}}{{s}_{{m}_{T}}}$$

with ${\mu }_{{m}_{T}}$ and ${s}_{{m}_{T}}$ being suitable standardization quantities.^5^ The limiting null distribution of ${\widehat{Z}}_{i,T}$ is standard normal.

In the panel setting, Presno et al. (2022) rely on the average of the individual test statistics, and the null of joint stationarity is tested through the following nonparametric panel stationarity (*NPS*) test statistic:

6 $${\overline{Z} }_{T}=\frac{\sum_{i=1}^{N}{\widehat{Z}}_{i,T}}{N}$$

Presno et al. (2022) propose a bootstrap-based implementation of the test. We shall also rely on the bootstrap to carry out both the individual and panel stationarity tests (the algorithms employed and related technical details may be found in Presno et al. 2022).

## Empirical analysis

### The dataset

We analyze monthly data of nine energy consumption sources in the US, namely coal, natural gas, petroleum, nuclear electric power, hydroelectric, geothermal, biomass, solar, and wind energy consumption. The study period spans from January 1973 to July 2022, except for solar and wind sources which begin in January 1989 due to data availability issues. The data source is the US Energy Information Administration (US-EIA) and the unit of measure is Quadrillion Btu. For the individual analysis we consider the nine series, whereas for panel analysis the solar and wind energy consumption series will be excluded with a view to maintaining a balanced panel.

As an overview, in the year 1973 around 93% of total primary energy consumption in the US came from fossil fuels,^6^ whereas that percentage dropped to 79% by 2021. Those 14 percentage points were replaced by nuclear electric power and renewable energy consumption, with the latter reaching a 12.4% share in 2021 (from an initial 6% in 1973). Efforts at both the state and federal levels to develop renewable energies in the US have clearly contributed to that increase in renewables.^7^ Among others, these actions included eliminating subsidies for nuclear and fossil energies, setting up renewable energy and portfolio standards, net metering programs, and production, investment, and sales tax credits. However, that percentage is a far cry from other geographic areas like the European Union, where around 22% of energy consumed in 2021 was generated from renewable sources. For its part, nuclear represented more than 8% of total primary energy consumption in the US in 2021.

The above monthly time series clearly exhibit seasonality. Table 2 reports the results of several seasonality tests (including nonparametric Kruskal–Wallis and parametric F-tests for seasonality, assuming stable and moving seasonality, respectively), which clearly indicate the presence of seasonal patterns in the individual series and also in the following four aggregates (obtained as sums of sources): total fossil fuel (coal + natural gas + petroleum) consumption, non-renewable (fossil fuels + nuclear electric power) energy consumption, renewable (hydroelectric + geothermal + biomass) energy consumption, and total (renewable + non-renewable) energy consumption, for the period 1973:01–2022:07. As for these aggregates, and for the sake of comparability with panel results, we did not consider wind and solar energy consumption since data were not available for those two sources on the initial period (1973:01–1988:12).

**Table 2:** Results of seasonality testing

Source\ Test	Kruskal–Wallis Chi-squared test	F-test (stable seasonality)	F-test (moving-seasonality)
Coal consumption (1973:01–2022:07)	494.049^c^ (*p* = 0.00)	252.536^c^ (*p* = 0.00)	6.791^c^ (*p* = 0.00)
Natural gas consumption (1973:01–2022:07)	517.235^c^ (*p* = 0.00)	670.216^c^ (*p* = 0.00)	4.078^c^ (*p* = 0.00)
Petroleum consumption (1973:01–2022:07)	357.154^c^ (*p* = 0.00)	64.171^c^ (*p* = 0.00)	1.528^b^ (*p* = 0.015)
Total fossil consumption (1973:01–2022:07)	499.372^c^ (*p* = 0.00)	342.558^c^ (*p* = 0.00)	4.694^c^ (*p* = 0.00)
Nuclear electric power consumption (1973:01–2022:7)	464.599^c^ (*p* = 0.00)	173.015^c^ (*p* = 0.00)	7.726^c^ (*p* = 0.00)
Non-renewable consumption (1973:01–2022:07)	497.382^c^ (*p* = 0.00)	352.214^c^ (*p* = 0.00)	2.335^c^ (*p* = 0.00)
Hydroelectric power consumption (1973:01–2022:07)	468.994^c^ (*p* = 0.00)	212.034^c^ (*p* = 0.00)	2.253^c^ (*p* = 0.00)
Geothermal energy consumption (1973:01–2022:07)	232.888^c^ (*p* = 0.00)	17.443^c^ (*p* = 0.00)	9.406^c^ (*p* = 0.00)
Biomass energy consumption (1973:01–2022:07)	286.418^c^ (*p* = 0.00)	32.827^c^ (*p* = 0.00)	5.646^c^ (*p* = 0.00)
Solar energy consumption (1989:01–2022:07)	383.594^c^ (*p* = 0.00)	1569.014^c^ (*p* = 0.00)	1.807^c^ (*p* = 0.006)
Wind energy consumption (1989:01–2022:07)	78.661^c^ (*p* = 0.00)	3.344^c^ (*p* = 0.00)	10.674^c^ (*p* = 0.00)
Renewable energy consumption (1973:01–2022:07)	438.844^c^ (*p* = 0.00)	152.212^c^ (*p* = 0.00)	1.190 (*p* = 0.186)
Total energy consumption (1973:01–2022:07	494.810^c^ (*p* = 0.00)	353.330^c^ (*p* = 0.00)	2.063^c^ (*p* = 0.00)

^a, b, c^ indicate significance at 10%, 5% and 1%, respectively

Because of the above, strong seasonal patterns, the series were de-seasonalized before proceeding with the analysis. We employed the TRAMO/SEATS software,^8^ which relies on a parametric seasonal adjustment based on seasonal ARIMA models and spectral analysis methods. This practice of de-seasonalizing has been common in previous papers employing quarterly or monthly information (e.g., Apergis and Tsoumas 2011, 2012; Golpe et al. 2012; Mishra and Smyth 2014, among others) and aims at alleviating the bias that strong seasonal behavior could cause on the stationarity tests.

### Results of stationarity analysis

Tables 3, 4, 5 report the results of stationarity testing,^9^ applied to the series previously adjusted for seasonality and converted to logarithms.

**Table 3:** Individual nonparametric stationarity testing results. Aggregate series

Source	Obs. NPS test statistic (*p*-value)
Total fossil fuels consumption (1973:01–2022:07)	0.869 (*p* = 0.547)
Non-renewable energy consumption (1973:01–2022:07)	0.846 (*p* = 0.532)
Renewable energy consumption (1973:01–2022:07)	0.668 (*p* = 0.151)
Total energy consumption (1973:01–2022-07)	0.838 (*p *= 0.509)

B = 10,000 bootstrap resamples were employed to compute the *p*-values.

**Table 4:** Panel nonparametric stationarity testing results

Panel	Obs. NPS test statistic (*p*-value)
Fossil fuels consumption (1973:01–2022:07)	0.292 (*p* = 0.605)
Non-renewable energy consumption (1973:01–2022:07)	3.014^b^ (*p *= 0.019)
Renewable energy consumption (1973:01–2022:07)	2.161^b^ (*p* = 0.036)
Total energy consumption (1973:01–2022-07)	3.693^c^ (*p* = 0.001)

**Table 5:** Individual nonparametric stationarity testing results

Source \ Treatment	DESEASONALIZED (TRAMO)
	Obs. NPS test statistic (*p*-value)
Coal (1973:01–2022:07)	-1.375 (*p* = 0.959)
Natural gas (1973:01–2022:07)	1.536^a^ (*p* = 0.054)
Petroleum (1973:01–2022:07)	1.339 (*p* = 0.355)
Nuclear (1973:01–2022:07)	6.131^c^ (*p* = 0.000)
Hydroelectric (1973:01–2022:07)	0.781 (*p* = 0.203)
Geothermal (1973:01–2022:07)	3.482^c^ (*p *= 0.001)
Biomass (1973:01–2022:07)	0.149 (*p* = 0.780)
Solar (1989:01–2022:07)	-2.189 (*p* = 0.999)
Wind (1989:01–2022:07)	-1.298 (*p* = 0.970)

#### Aggregate energy consumption and panel analysis

Starting with aggregated energy consumption (Table 3), it is observed that all the deseasonalized series (fossil, non-renewables -including nuclear-, renewables, and total energy consumption) show stationary behavior. This result matches previous research (Hasanov and Telatar 2011; Demir and Gozgor 2018) in aggregate energy consumption and may be taken as preliminary evidence of stationarity in the disaggregated individual series (keep in mind that unit root is a dominant character). However, as also pointed out by Yang (2000), aggregate energy data do not fully capture the extent to which the countries depend on the various energy sources. In addition, scalar stationarity tests usually exhibit low power, especially in short series. These considerations led us to employ panel stationarity testing with a view to both gathering further evidence and obtaining more powerful results.

Table 3 reports the results of panel stationarity testing, with the panels considered being fossil fuel consumption (coal, petroleum, natural gas), non-renewables (coal, petroleum, natural gas, nuclear electric power), renewables (hydroelectric, geothermal, and biomass energy consumption), and total energy consumption (coal, petroleum, natural gas, nuclear, hydroelectric, geothermal, biomass).

The results of nonparametric panel stationarity tests in Table 4 indicate that stationarity fails to be rejected for the non-renewables panel, which only includes fossil fuels energy consumption; however, the inclusion of energy consumption from nuclear sources in the panel leads to reject the null of stationarity. Meanwhile, the null of stationarity is rejected at 5% significance level for the panel of renewable energy sources of consumption and, consistently with the conclusions obtained in previous subpanels, stationarity is again rejected for the panel of seven (renewable + non-renewable) series. Although it is often argued that stationarity tests are biased in favor of the null hypothesis of stationarity, the complete panel analysis would lead us to reject that hypothesis in this case. Also, it seems that conclusions depend on the specific source of energy consumption analyzed, which points to the convenience of studying the various sources of energy consumption separately. This conclusion is in accordance with Lean and Smyth (2009), who strongly defend –in their analysis of petroleum consumption– the convenience of distinguishing among different forms of energy.

#### Stationarity analysis by source

The above results suggest the convenience of also analyzing stationarity individually for each consumption source, in order to detect specific series that may be responsible for panel non-stationarity. Table 5 includes the results of individual stationarity testing. Stationarity is rejected, at 1% significance, in nuclear and geothermal energy consumption. The same conclusion is obtained for natural gas series at 10% significance, but not at the customarily used 5%.

As an additional safeguard, we applied Hommel’s (1988) modified Bonferroni testing procedure, with the aim of both controlling for the multiplicity and familywise error rate^10^ (FWER) of the testing problem and identifying those series in the panel that exhibit non-stationarity. For this we employed the implementation proposed by Landajo and Presno (2022), who adapted to panel stationarity the proposal of Hanck (2013) and Hanck and Czudaj (2015) for unit root testing. The method builds on Hommel’s method to detect specific series in the panel for which the alternative hypothesis (i.e., integratedness) appears to be true.

For a significance level α = 0.05, and from the *p*-values in Table 5, we constructed Table 6, which allows us to detect specific sources in the panels for which the null is rejected. Again, the results point to non-stationarity for nuclear and geothermal energy consumption, confirming the conclusions of the univariate tests in Table 5 above.

**Table 6:** Summary of conclusions of Hommel´s procedure (α = 0.05)

Panel	Conclusions
Overall panel. *N* = 7	Stationarity rejected for nuclear and geothermal energy consumption
Non-renewable panel *N* = 4	Stationarity rejected for nuclear energy consumption
Renewable panel *N* = 3	Stationarity rejected for geothermal energy consumption

#### Comparative study and discussion

Several previous papers have studied the US oil consumption series. Lean and Smyth (2009) evaluate the long memory of disaggregated US data, reaching different conclusions depending on the sector under study. Apergis and Payne (2010) focus on US states, concluding that the null hypothesis of a unit root in petroleum consumption must be rejected for most of the series they analyze. Our results also indicate stationarity of aggregate US oil consumption. This conclusion is in line with Solarin and Lean (2016), who find linear behavior in the oil series and conclude stationarity in the case of US consumption. This would imply that shocks are temporary and energy conservation and demand management policies (such as environmental protection strategies, carbon emission restrictions, and fuel economy standards) meant to curb oil consumption only have transitory effects, being ineffective in the long term as it is likely that the series returns to its trend path. Along this line, Solarin and Lean (2016) recommend policymakers to implement short-term strategies such as gradual reductions in oil subsidies and increases in the taxes on oil consumption. Another implication is that shocks to petroleum consumption would not be transmitted (via flow-on effects) to the macroeconomic sectors. Stationarity also implies, in terms of forecasting, that the past behavior of the series can be usefully exploited for prediction purposes.

Coal consumption is classified as stationary, so energy conservation policies (with their focus on permanent reductions in coal consumption) would exclusively have transitory effects since the evolution of the series will tend to return to its trend. Apergis et al. (2010a) also concluded panel stationarity in their study for the 50 US states.

Natural gas consumption would exhibit non-stationarity at 10% significance. However, the tests would indicate stationarity at 5% significance, so the evidence is ambiguous in this case. There has been considerable debate in the literature on the linearity of this series. Apergis et al. (2010b), in their analysis of US states, applied panel tests and observe that conclusions about the stochastic properties of natural gas consumption would change once endogenously determined structural changes are allowed for, favoring the hypothesis of stationarity. Aslan (2011), also for the US states, finds nonlinearity in around 60% of the series and reports roughly 50% of stationary and non-stationary states. In their analysis on persistence in natural gas consumption, Golpe et al. (2012) find a non-stationary process in the regime, characterized by positive variations in consumption; they also warn that, due to contradictory conclusions found in the literature on long memory properties, policy should be careful. Mishra and Smyth (2014) emphasize that conditional heteroscedasticity is particularly problematic in higher frequency (e.g., monthly) data, concluding stationarity in monthly US natural gas consumption. Regarding this, the non-parametric methodology we rely on in this paper automatically incorporates the possibility of non-linearities and should be robust in this sense. Simulations by Landajo and Presno (2013) and Presno et al. (2022) showed the good behavior of the test in nonlinear time series models, including bilinear and conditionally heteroscedastic processes.

As for nuclear consumption, Zhu and Guo (2016) classify 27 countries into four clusters defined according to their nuclear energy consumption per capita, including the US (together with around 50% of the countries in their sample) in the “upper-middle” cluster; they find that, for that specific cluster, the effects of shocks to nuclear energy consumption are permanent. In our case, stationarity is also rejected for US nuclear energy consumption, implying that random shocks (i.e., regulatory changes) may produce permanent deviations from the original target levels, and that these changes possibly have a serious effect on the real economy.

In the field of renewable sources, and in contrast to Aydin and Pata (2020) who conclude that most renewable energy consumption series in the US exhibit unit roots, our research indicates that only geothermal consumption would be non-stationary. More precisely, we coincide with their conclusions on the stationarity of hydropower and non-stationarity of geothermal whereas our conclusions differ from theirs for the biomass, solar, and wind series. In this regard, Barros et al. (2013), analyzing persistence and long-memory in these series, also find non-stationary but mean-reverting^11^ behavior in hydropower, solar, and wind series, although their estimate of the fractional integration parameter in the geothermal consumption series is on the boundary between stationarity and non-stationarity. The wood consumption series would be clearly stationary according to their analysis.

In recent years geothermal energy consumption has lost relative weight within renewable sources. In our analysis, the null of stationarity is rejected, implying that shocks have a permanent effect.

According to our results, hydropower is stationary. The same conclusion was arrived at by other studies carried out for several countries (e.g., Wang et al. 2016, for Japan, Aydin and Pata 2020, for the US). In this context, Pata and Aydin (2020) conclude that hydropower energy consumption has no effect on environmental pressure and economic growth in the US, and it is not a major problem for US renewable energy policies.

### Cyclical behavior in energy consumption

As a complement to the above stationarity analysis, in this section we include a brief study of cycles^12^ in the sources of energy consumption in the US. We begin by applying the turning- points-dating method, proposed by Bry and Boschan (1971), to identify troughs and peaks of the cycles in the various energy sources (the time series of wind and solar energy consumption were shorter than the rest of the panel, so we opted for not including them in the cycle analysis in order to keep the panel balanced and facilitate comparisons). Then, we will study the degree of synchronization both among the cycles identified for the various sources, and between each source and the business cycle expansions and contractions, as estimated by the NBER.

In their seminal paper, Burns and Mitchell (1946) define several concepts linked to cycles, such as peak (the high point of an expansion period) and trough (the worst moment in a recession), which are relevant to determining cycle length. On that basis, Bry and Boschan (1971) (BB hereafter) proposed a non-parametric procedure that is commonly applied by the NBER to determine cycle dating.^13^ In its implementation, and with a view to suppressing noise sources unrelated to business cycles, the algorithm requires to specify a time window (on which to identify the turning points), a minimum time length for the duration of each phase, and the minimum duration of the complete cycle. Given the monthly periodicity of the data,^14^ we followed Brown (2017) and considered a 12-month window, with each phase lasting at least 6 months, and each cycle covering 24 months. Finally, to overrule the minimum phase restriction, we set the “threshold parameter” at 25%.

Table 7 summarizes the main characteristics of the cycles identified by the above method and the end state obtained for the various sources. Results indicate that, except for coal and nuclear consumption, all the sources were in an expansion phase at the final period. More precisely, after a brief drop at the beginning of 2020 (coinciding with the arrival of the COVID pandemic^15^), petroleum went on with the expansive phase that started by the end of the year 2012. Natural gas consumption also had an expansive phase from the end of 2020, whereas coal was the only fossil fuel that showed a contraction phase, since the beginning of 2021 (indeed, coal was in a recessive phase since early 2018 and only recovered briefly around the middle of 2020, along with the COVID^16^ outburst, but then it came back to a recessive phase). Nuclear has been in a recessive stage since 2017. As for renewable sources (geothermal, biomass, and hydroelectric power consumption), they have been in an expansive phase since 2019, 2020, and 2021, respectively. In line with these results, Yasmeen et al. (2022) conclude that, comparing renewable and non-renewable energy consumption, the latter was more affected by the COVID lockdown.

**Table 7:** Characteristics of the cycles, by energy source. Period 1973:01–2022:07

Source	Volatility	Number of cycles	Duration (months)		Final state
			Expansion	Recession	
Coal	0.48	11	33.72	18.58	Recession Last peak: 2021:02
Natural gas	0.50	9	32.70	26.60	Expansion Last trough: 2020:11
Petroleum	0.50	8	34.89	31.11	Expansion Last trough: 2020:04
Nuclear	0.42	5	76.83	22.17	Recession Last peak: 2017:10
Hydroelectric	0.49	9	23.50	39.89	Expansion Last trough: 2021:04
Geothermal	0.43	9	45.9	16.33	Expansion Last trough: 2019:11
Biomass	0.40	6	67.86	19.83	Expansion Last trough: 2020:04

The table summarizes estimated volatility (measured by the standard deviation of the state), number of cycles, and their average duration (in months)

Once the existence of certain patterns in the cycles of the various sources of energy consumption has been established, we shall analyze potential binary relationships between the phase states of different sources of energy consumption, and also between each source and the US business cycle expansions/contractions as called by the NBER. This will allow us to examine the possibility that they may be coordinated (i.e., so-called synchronization). To that end, we rely on the concordance index in its bivariate form (as defined by Harding and Pagan 2002) and the test for no concordance (Harding and Pagan 2006).

Harding and Pagan (2002) define the concordance index for any two regions *i* and *j* (in our case, two sources) as follows:

7 $${I}_{i,j}={T}^{-1}\left[{\sum }_{t=1}^{T}\left({S}_{it}{S}_{jt}\right)+{\sum }_{t=1}^{T}\left({1-S}_{it}\right)\left(1-{S}_{jt}\right)\right]$$

where *T* is sample size, ${S}_{it}$ (or ${S}_{jt}$) is a dummy variable taking value 1 when the *i*-th (or the *j*-th) source is in an expansion phase and 0 when it is in a recession phase. ${I}_{i,j}$ measures the proportion of time that the two sources (or one source and NBER´s business cycle) are in the same phase. This indicator is intuitive, but it has the drawback of not providing a statistical measure of the significance of comovements. Thus, Harding and Pagan (2006) proposed another measure, namely ${\rho }_{{S}_{ij}}$, which is based on the correlation between ${S}_{jt}$ and ${S}_{it}$ and may be estimated from the following regression:

8 $${\sigma }_{{S}_{it}}^{-1}{\sigma }_{{S}_{jt}}^{-1}{S}_{it}=\alpha +{\rho }_{{S}_{ij}}{\sigma }_{{S}_{it}}^{-1}{\sigma }_{{S}_{jt}}^{-1}{S}_{jt}+{\varepsilon }_{t}$$

where ${\sigma }_{{S}_{it}}$ a and ${\sigma }_{{S}_{jt}}$ are the standard deviations of the time series ${S}_{it}$ and ${S}_{jt}$, respectively. This allows the null ${\rho }_{{S}_{ij}}=0$ (i.e., no concordance between the series) to be readily tested (the Newey-West HAC covariance –with Bartlett weights– estimator is usually employed).

Table 8 includes the results of concordance analysis between states of the various sources of consumption. The results of concordance analysis between NBER´s business cycle expansions/contractions and consumption source states^17^ appear in Table 9. As a rule, in both Tables 8 and 9 we observe that pairwise correlations are smaller than those obtained from the concordance index, indicating that the concordance between sources of consumption as measured by ${I}_{i,j}$ is inflated by the values of the mean^18^ (namely, ${\upmu }_{S}=E\left({S}_{t}\right)$; see the last row of Table 8). Significant pairwise correlations are observed between the states of petroleum consumption and all the sources except coal consumption; looking at the signs, we find negative correlations between petroleum consumption and nuclear, geothermal, and biomass sources. In addition, geothermal exhibits correlation with all the sources except natural gas and nuclear, with only the correlation with petroleum being negative.

**Table 8:** Concordance indices and correlations of states between sources

	Coal	Natural gas	Petroleum	Nuclear	Hydroelectric	Geothermal	Biomass
Coal	…	0.580	0.541	0.585	0.514	0.662	0.575
Natural gas	0.144 (1.597)	…	0.669	0.568	0.454	0.467	0.487
Petroleum	0.073 (0.796)	0.336^c^ (3.967)	…	0.435	0.566	0.408	0.452
Nuclear	0.035 (0.455)	0.082 (1.101)	-0.161^b^ (-2.282)	…	0.455	0.634	0.718
Hydroelectric	0.084 (0.986)	-0.073 (-0.827)	0.145^a^ (1.695)	0.036 (0.343)	…	0.529	0.476
Geothermal	0.212^c^ (2.758)	-0.117 (-1.590)	-0.212^c^ (-2.898)	-0.018 (-0.199)	0.171^b^ (2.440)	…	0.734
Biomass	0.001 (0.020)	-0.086 (-1.259)	-0.129^a^ (-1.834)	0.149 (1.512)	0.079 (1.128)	0.222^b^ (2.430)	…
${\widehat{\upmu }}_{S}$	0.624	0.550	0.528	0.776	0.397	0.756	0.8

Above the diagonal: concordance index ${\widehat{I}}_{i,j}$. Below the diagonal: correlations of cycles $({\widehat{\rho }}_{{S}_{ij}})$ and robust *t*-statistics (null hypothesis: no correlation) in parentheses

The dependent variable in the regression is shown in columns; conclusions for the other case are identical

**Table 9:** Concordance indices and correlations of states between source and business cycle

	Coal	Natural gas	Petroleum	Nuclear	Hydroelectric	Geothermal	Biomass
${\widehat{I}}_{i,j}$	0.674	0.644	0.622	0.686	0.440	0.649	0.682
${\widehat{\rho }}_{{S}_{ij}}$	0.373^c^ (3.295)	0.489^c^ (5.311)	0.464^c^ (4.962)	-0.101 (-1.156)	0.083 (0.677)	-0.190^b^ (-2.504)	-0.198^c^ (-4.119)

Linear regression of source on business cycle. Robust *t*-statistics (null hypothesis: no correlation) in parentheses. The mean of business cycle is ${\widehat{\upmu }}_{S}$ =0.876

The above analysis can be extended in order to analyze concordance/correlation between business cycle and energy consumption source states.^19^ The results appear in Table 9 below (the model in this case is a simple linear regression of business cycle on each source), indicating statistically significant correlations between business cycle and energy source states, positive with fossil fuels (coal, natural gas, petroleum) but negative with two renewable sources (namely, geothermal and biomass energy consumption).

**Fig. 1:**
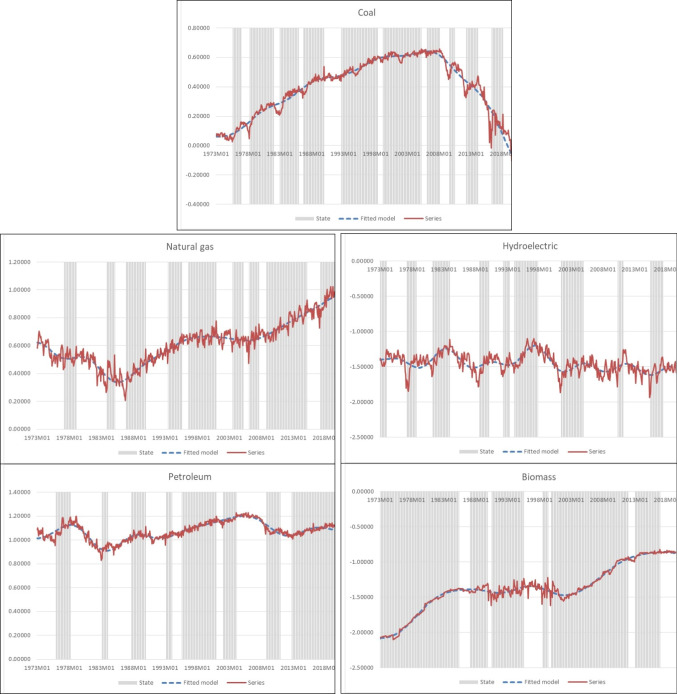
Observed time series (in logarithms, continuous line), fitted nonparametric models (dashed line), and cycle states (solid bars; shaded areas indicate expansion periods whereas recessions appear unshaded)

Figure 1 below summarizes the results of cycle analysis and the models fitted for five of the series classified as stationary according to our analysis.^20^

## Concluding remarks

We have analyzed stationarity in a group of nine energy consumption sources for the period 1973–2022. For many of these series, the presence of structural changes and non-linearities has been documented in previous research. Those factors are well-known to adversely affect the size and power of both unit-root and stationarity tests. Our study employs a nonparametric, bootstrap-based stationarity testing approach that avoids the need for prior model specification of the deterministic components of the series. The study was carried out both individually for each series and at the panel level.

The results of panel analysis find no statistically significant evidence against trend-stationarity in the group of energy consumption from fossil fuels, whereas the inclusion of nuclear energy consumption in the panel of non-renewable sources leads us to reject stationarity. At the same time, stationarity would be rejected for the panel of renewable sources. In line with these results, the same hypothesis is also rejected for the complete panel, which includes both renewable and non-renewable sources. These conclusions differ from the results for the series of aggregate energy consumption (obtained as the sum of all the individual series), possibly because of the lesser power of individual stationarity tests as compared with their panel counterparts.

Additionally, we have carried out a univariate stationarity analysis of the series, complemented with Hommel’s modified Bonferroni procedure, to detect specific units responsible for the rejection of stationarity at the panel level. In congruence with the results of the individual stationarity tests, stationarity is rejected for nuclear and geothermal energy consumption. This stresses the convenience of studying energy consumption disaggregated by source, since there may be significant changes in the conclusions, depending on the specific source of consumption considered.

Consistently with the above conclusions, the transitory nature (for all the energy sources studied excepting geothermal and nuclear consumption) of the effects of regulatory changes (i.e., shocks) entails that they may not have a relevant influence on any aspects (e.g., output and employment) of the real economy, which generally discourages implementation of demand stabilization policies in those sectors; otherwise said, these results suggest that US administration should not conduct needless interventions to induce changes in these energy sources.

However, according to our results, nonstationarity implies that supply, demand, and policy shocks may have permanent effects on geothermal and nuclear energy consumption. Therefore, energy promoting policies can be effectively implemented in these two cases. To the extent that these sectors are integrated with the overall economy, any shocks to them that are permanent in nature may be transmitted to other sectors.

From the standpoint of modeling and forecasting future energy consumption, the above results also indicate that past information may be usefully exploited to generate forecasts in all the series analyzed, with the exceptions of geothermal and nuclear energy consumption, where such efforts would be of little or no use.

Another aspect that may help us understand the dynamics of energy consumption in the US is the potential existence of cycles –peaks and troughs- in the series of consumption by source. We addressed this analysis by applying the Bry-Boschan procedure, which allowed us to detect those specific points for the various sources. In this regard, we have found that (with the exceptions of coal and nuclear) all the sources analyzed are in the final period of an expansion phase. As for nuclear consumption, our results seemingly match the revival of interest in nuclear power at the beginning of the 2000s (so-called *nuclear renaissance* in the US and fueled by the Energy Policy Act of 2005). The situation was more complex for nuclear in the second decade of this century, with serious challenges including the 2011 Fukushima Daiichi nuclear disaster and the surplus of natural gas utilities (after the rise of hydraulic fracturing and horizontal drilling) that fostered cancellations in some nuclear projects. More recent actions (e.g., the Nuclear Energy Innovation Capabilities Act of 2017, the Infraestructure Investment and Jobs Act of 2021, and the Inflation Reduction Act of 2022) may be helpful to revert this recessive phase. As a matter of fact, while nuclear energy remains a polarizing issue amongst public opinion, energy authorities have tended to incorporate a share of nuclear energy into their green energy goals.

Coal consumption peaked in the second half of the 2000s decade and has been largely replaced, mainly by natural gas. Other drivers were the closure of old plants and the passing of new, more stringent environmental regulations. However, at the beginning of the COVID pandemic in 2020, the second half of the year was interspersed with an expansive phase.

An analysis of synchronization between states also allowed us to detect significant pairwise correlations between the states of petroleum consumption and all the sources except coal consumption. The analysis reveals negative correlations with nuclear and two renewable sources (namely, biomass and geothermal energy consumption). Another source of energy consumption, correlated with all the sources except natural gas and nuclear, is geothermal consumption. Its correlation is positive with the other renewable sources (i.e., biomass and hydroelectric energy consumption).

An issue that has commanded particular interest in energy has been the analysis of causality relationships between energy consumption and GDP. We do not go deeper into this subject, but our analysis of correlations between the states of business cycle and energy sources reveals statistically significant correlations, positive with fossil fuels and negative with two renewable sources (namely, geothermal and biomass), whereas no statistically significant correlations are detected with nuclear and hydroelectric energy consumption states.

In the US, the fossil fuel share in consumption is nearly 80% and increased by about 2% in 2022 with respect to the previous year. This fact makes the American economy highly dependent on fossil fuels, with renewables finding it hard to increase their share. This stresses the importance of continuing with programs to promote innovation clusters devoted to making advances in clean energy, as well as increasing the funding for clean energy with a view to accelerating solutions meant to address climate change. It is also crucial to encourage both private and public sectors to work together on making investments in clean energy solutions. Research in areas such as long-duration energy storage, carbon capture systems, clean hydrogen, and advanced nuclear reactors will play a key role in properly addressing the climate goals. It is also urgent to accelerate the US energy transition to net zero emissions. Finally, effective coordination of energy policies at the federal, state, and local levels will also be crucial to simultaneously address climate change and boost the economy.

## Data Availability

The datasets used and/or analysed during the current study are available from the corresponding author.

## Footnotes

1 A complete, up-to-date review of stationarity analysis applied to energy consumption series may be found in Aydin and Pata (2020).

2 Smyth (2013) includes a survey of literature on the stochastic properties of energy consumption.

3 Congregado et al. (2012) analyzed coal consumption in the USA by employing unit root tests with endogenous structural breaks, concluding that a unit root exists in that series.

4 The test relies on the method of sieves, whereby model complexity increases with sample size at a suitably restricted rate in order to deliver a consistent test with asymptotically correct size. This differs from the Becker et al. (2006) proposal, which relies on a fixed parametric structure (not depending on sample size) to approximate the unknown trend function. More specifically, they consider two possibilities, so-called BEL1 (employing a single-frequency component of the classical Fourier expansion in sine and cosine functions) and BEL2 (which typically relies on employing only the first two frequencies of the Fourier expansion, with a view to avoiding power loss). Landajo and Presno (2013) include extensive Monte Carlo simulations comparing the finite-sample performance of the three approaches.

5 $${\mu }_{{m}_{T}}= \sum_{j={m}_{T}+1}^{\infty }{(j\pi )}^{-2}$$,$${s}_{{m}_{T}}^{2}=2\sum_{j={m}_{T}+1}^{\infty }{(j\pi )}^{-4}$$, and$${s}_{{m}_{T}}=+\sqrt{{s}_{{m}_{T}}^{2}}$$.

6 More specifically, petroleum and natural gas went from 46% and almost 30%, respectively, of total primary energy consumption in 1973, to 36% and 32% in 2021.

7 See Herath and Tyner (2019).

8 To check the robustness of results when different methods of de-seasonalizing were employed we also applied the X-12-ARIMA seasonal adjustment method. The conclusions were virtually identical to those reported in this paper. The results are available from the authors upon request.

9 A similar non-parametric approach was employed by Landajo *et. al.* (2021) to study the prices of energy commodities, whereas Landajo and Presno (2022), relying on a parametric approach, analyzed the prices of renewable commodities.

10 I.e., the probability of incorrectly rejecting at least one true (individual time series) null hypothesis at the specified α level.

11 According to Barros et al. (2013), the estimated values for the fractional differencing parameter exceed 0.5 but are below 1.0, which implies that shocks have persistent effects, but the processes would eventually return to their long-run mean.

12 Other papers that have applied this methodology to analyze environmental variables are Zerbo and Darne (2019) and Churchill et al. (2020).

13 Although some of the series were classified as integrated in the previous section, we relied on the classical definition of cycle, which applies to the behavior of the business cycle in levels (i.e., the undifferenced series). As pointed out by Harding and Pagan (2002): “there is no need to perform a detrending operation to analyze the business cycle. Indeed, the act of removing a stochastic trend actually eliminates one of the major driving forces of the business cycle and is therefore to be avoided”.

14 Pagan (2010) considered a 5-month window and a minimum phase and cycle length of 6 and 15 months, respectively, whereas Brown (2017) asserts that setting these parameters too short could lead to false positive indications of recessions. We applied both approaches in our analysis: the results (available on request) indicate that the turning points identified by applying Pagan’s proposal to our dataset were the same as those of Brown’s method plus a few additional ones.

15 Total primary energy consumption fell by around 7.5% in 2020 compared to 2019, with the drop in oil consumption (around 12%, mainly because of a decrease in travel) being more pronounced. However, in 2021 there was an increase of 5.3% (around 9% in the case of oil) in total primary energy consumption in comparison with the previous year, with the pre-pandemic levels of 2019 not yet reached in 2021.

16 Energy consumption rebounded in late 2020 and throughout 2021, briefly returning coal to economic favor. More specifically, the Energy Information Administration estimated that in 2021 electricity generation from coal was 17% higher than the previous year. However, 12.6 gigawatts of coal capacity were set to be retired in 2022.

17 Brown (2017) compares the turning points identified by the Bry-Boschan method and NBER, concluding that they are similar for most recessions.

18 A case in point is nuclear and biomass where, despite the high *I*_*i*_*,*_*j*_ value (0.718), the correlation between them is not statistically significant.

19 Although the focus of this paper is on raw statistical association, the analysis may also be extended to analyze causality in the relationship between energy consumption and growth, as suggested by the energy conservation, growth, feedback, and neutrality hypothesis. See Sari et al. (2008) for a literature review on this topic, and more recent contributions by Al-Mulali et al. (2013), Omri et al. (2015), and Mahalaingam and Orman (2018), among others.

20 For the remaining two stationary series the cycle analysis was omitted as their range of observation was shorter; in the case of the series classified as integrated, model estimation would require previous differencing of the series.
